# Lung-DT: An AI-Powered Digital Twin Framework for Thoracic Health Monitoring and Diagnosis

**DOI:** 10.3390/s24030958

**Published:** 2024-02-01

**Authors:** Roberta Avanzato, Francesco Beritelli, Alfio Lombardo, Carmelo Ricci

**Affiliations:** 1 Department of Electrical, Electronic and Computer Engineering, University of Catania, Viale Andrea Doria, 95125 Catania, Italy; roberta.avanzato@unict.it (R.A.); alfio.lombardo@unict.it (A.L.); or carmelo.ricci@cnit.it (C.R.); 2 National Inter-University Consortium for Telecommunications (CNIT), RU of Catania, 43124 Parma, Italy

**Keywords:** Digital Twin, IoT sensors, image processing, lung healthcare, smart healthcare, convolutional neural network, deep learning

## Abstract

The integration of artificial intelligence (AI) with Digital Twins (DTs) has emerged as a promising approach to revolutionize healthcare, particularly in terms of diagnosis and management of thoracic disorders. This study proposes a comprehensive framework, named Lung-DT, which leverages IoT sensors and AI algorithms to establish the digital representation of a patient’s respiratory health. Using the YOLOv8 neural network, the Lung-DT system accurately classifies chest X-rays into five distinct categories of lung diseases, including “normal”, “covid”, “lung_opacity”, “pneumonia”, and “tuberculosis”. The performance of the system was evaluated employing a chest X-ray dataset available in the literature, demonstrating average accuracy of 96.8%, precision of 92%, recall of 97%, and F1-score of 94%. The proposed Lung-DT framework offers several advantages over conventional diagnostic methods. Firstly, it enables real-time monitoring of lung health through continuous data acquisition from IoT sensors, facilitating early diagnosis and intervention. Secondly, the AI-powered classification module provides automated and objective assessments of chest X-rays, reducing dependence on subjective human interpretation. Thirdly, the twin digital representation of the patient’s respiratory health allows for comprehensive analysis and correlation of multiple data streams, providing valuable insights as to personalized treatment plans. The integration of IoT sensors, AI algorithms, and DT technology within the Lung-DT system demonstrates a significant step towards improving thoracic healthcare. By enabling continuous monitoring, automated diagnosis, and comprehensive data analysis, the Lung-DT framework has enormous potential to enhance patient outcomes, reduce healthcare costs, and optimize resource allocation.

## 1. Introduction

Early diagnosis of lung pathologies is crucial for improving patient prognosis. So far, chest X-rays are one of the most common imaging modalities used for diagnosing lung pathologies. However, diagnosing lung pathologies based on chest X-rays alone can be challenging and subjective, especially for diseases with similar symptoms.

In recent years, deep learning (DL) has shown significant potential in enhancing the diagnosis of lung pathologies based on chest X-rays. DL, a branch of artificial intelligence, utilizes artificial neural networks to learn complex patterns from data. DL systems for lung pathology recognition based on chest X-rays can be classified into two main categories:

- Detection Systems: these systems are designed to identify the presence of anomalies in chest X-rays.
- Classification Systems: these systems are designed to classify anomalies in chest X-rays based on the type of lung pathology.

Common DL techniques used for lung pathology recognition based on chest X-rays include

- Convolutional Neural Networks (CNNs): CNNs are a class of artificial neural networks designed for image processing. They have been successfully employed in order to detect anomalies in chest X-rays, including lung nodules, lung infiltrates, and pneumonia.
- Recurrent Neural Networks (RNNs): RNNs are a class of artificial neural networks designed for processing sequence data. RNNs have been successfully used to identify anomalies in chest X-rays that develop over time, such as the progression of lung cancer, for example.
- Generative Adversarial Networks (GANs): GANs are a class of artificial neural networks designed to generate realistic data. GANs have been successfully used for synthesizing chest X-ray images containing anomalies, thus improving the training of DL systems.

Numerous studies have demonstrated the effectiveness of DL in analyzing chest X-rays, achieving remarkable results in the detection and classification of lung nodules, pneumonia, and other thoracic pathologies. For example, Wang et al. developed a DL-based system for lung nodule detection with promising results [1]. Similarly, Kermany et al. demonstrated the effectiveness of DL in classifying pneumonia from chest X-rays [2].

These advancements highlight the transformative potential of DL in chest X-ray analysis. As research efforts continue to refine DL algorithms and expand their range of application, this powerful technology is poised to revolutionize the detection, diagnosis, and treatment of lung diseases, ultimately improving patient outcomes.

Despite remarkable progress, several challenges are faced, impeding full realization of the DL potential in chest X-ray analysis. These challenges include the need for large sets of high-quality annotated image data, ensuring the generalizability of DL models to diverse patient populations, and addressing ethical and regulatory considerations associated with deploying AI in healthcare.

To address these challenges, research efforts should focus on creating comprehensive image datasets, developing robust DL models capable of effectively handling real clinical scenarios, and establishing clear guidelines for the ethical and responsible use of AI in healthcare.

The healthcare landscape is undergoing a profound transformation driven by the convergence of advanced technologies, including not only DL but also the Internet of Things (IoT) and Digital Twins (DTs). These innovations offer enormous potential to revolutionize healthcare delivery in general and, particularly, the field of monitoring lung pathologies.

IoT sensors integrated into wearable devices connected to the Internet enable real-time monitoring of physiological parameters such as heart rate, blood pressure, respiratory rate, and X-ray images of various organs. These continuous data streams enable proactive healthcare interventions, allowing for early detection of potential complications and facilitation of remote patient monitoring.

By providing virtual representations of physical objects or systems, DTs offer a holistic approach to monitoring lung pathologies. By integrating data from IoT sensors, clinical records, and other relevant sources, DTs can provide a comprehensive view of an individual’s lung health, allowing for early detection of subtle changes and personalized treatment recommendations.

To fully harness the potential of these technologies, a unified platform is essential to seamlessly integrate AI-enhanced image analysis, IoT sensor data, and DT simulations. This platform would provide clinicians with a centralized hub for real-time information, enabling them to make informed decisions and optimize patient care.

In particular, lung pathology monitoring based on DL, IoT, and DTs offers numerous benefits, including

- Early and Accurate Detection of Lung Pathologies: AI-enhanced image analysis systems can support radiologists in identifying anomalies in chest X-rays, providing technological assistance for a quick and precise diagnosis;
- Personalization of Diagnosis and Treatment: data collected from IoT sensors and DTs can be used to develop personalized diagnoses and treatment plans that are more effective and cost-efficient;
- Improvement in Patients’ Quality of Life: remote monitoring systems enable patients to receive care from home, improving their quality of life and reducing healthcare costs.

This study brings forward a proposal for the application of DT technology in the classification of lung pathologies, using chest X-ray images as input. The main objectives of this study, which also constitute the innovative aspect compared to the state of the art described in Section 2, are as follows:

- Implementation of a case study that includes a proof of concept of a Lung-DT based on a microservices architecture characterized by an artificial intelligence component.
- The proposed Lung-DT architecture is designed to acquire various input signals, including chest X-ray images (the subject of our study) and blood oxygen saturation (to be integrated later), allowing for a more comprehensive evaluation of lung conditions.
- Extension of the second-level architecture previously proposed in [3]. This extension involves the integration of DTs related to other organs in order to define the presence of pathologies. The use of information from different organs enables more comprehensive detection and classification of pathological conditions.
- Accurate classification of lung pathologies into five categories to contribute to a more detailed and specific diagnosis.
- Expansion of the neural network’s training and validation dataset by integrating two public datasets. This integration aims to enhance the predictive capacity of the model, improving its adaptability to a wider range of pathologies.
- Use of a third dataset completely unknown to the neural network to perform additional testing. This step is aimed at confirming the robustness of the model by evaluating its performance on novel data.

The rest of the paper is structured as follows: Section 2 provides an overview of the state of the art in terms of DT-based systems applied to lung pathology recognition using chest X-ray images and DT architecture applied to the lung. The architecture and implementation of the Lung-DT, and, more generally, the healthcare DT platform, are described in Section 3. The setup description of the Lung-DT is provided in Section 4. This section outlines the dataset made up of four lung pathologies, including the “normal” classification, the neural network model, and the obtained results. Section 5 provides the comparison of the research present in the state of the art and the Lung-DT proposed in this paper. A paragraph related to ethical aspects and theoretical examples has been inserted in Section 6. Finally, Section 7 draws the conclusions of this study.

## 2. Related Work

The field of medical diagnostics is undergoing a revolution thanks to the advanced use of artificial intelligence techniques applied to the analysis of chest X-ray images. This chapter aims to examine the latest methodologies proposed in the scientific literature for the diagnosis of lung diseases through the use of artificial neural networks and machine learning algorithms.

In recent years, especially during and after the COVID-19 pandemic, numerous DL methods have been proposed for the purpose of the detection of lung diseases employing X-ray images. Some of these studies [4,5,6] adopt a simpler approach, directly classifying chest X-ray images into a set of categories corresponding to each lung disease, without preprocessing and feature extraction. These approaches are generally simpler and computationally efficient. Other studies [7,8,9,10,11,12,13,14,15], however, use more complex approaches typically consisting of two phases: feature extraction and classification. In the feature extraction phase, DL algorithms extract relevant features from chest X-ray images, and, in the classification phase, these features are used to classify images into different categories of lung diseases. Compared to the first approach, these methods are computationally more complex and slower. Finally, some studies [16,17] combine both approaches to conduct more comprehensive research and compare the performance of various systems in terms of accuracy.

In [4], a self-supervised deep neural network is proposed, using chest X-rays as an efficient and widely available method for classifying respiratory diseases. The self-supervised deep neural network, pretrained on an unlabeled dataset, utilizes contrastive learning to transfer learned representations to subsequent classification tasks. The results demonstrate that this approach achieves competitive performance without requiring large amounts of labeled training data. This study adopts a straightforward approach without a separate feature extraction phase. Similarly, in [5], the authors present a hybrid architecture that combines contrast-limited adaptive histogram equalization (CLAHE) with a deep convolutional network for the classification of lung images into different pathology classes. By using chest X-ray images, the authors claim that this method outperforms traditional approaches by 20% in terms of accuracy, demonstrating its effectiveness in early diagnosis and categorization of lung diseases. In [6], the authors propose a classification algorithm based on the SqueezeNet neural network to distinguish between chest X-rays of individuals with and without lung diseases. Using a dataset of chest X-ray images, the model is tested with an accuracy of 94%, demonstrating its ability to discriminate between “Normal” and “Pneumonia” images.

In [7], a hybrid approach with modular neural networks is represented, suggesting the use of modular artificial neural networks integrated with fuzzy logic for the diagnosis of lung diseases. This method focuses on the analysis of digitized chest X-ray images, using descriptors such as the gray-scale histogram and gray-level co-occurrence matrix. The use of a multi-objective genetic algorithm to reduce features enables the creation of an optimized neuro-fuzzy classifier, demonstrating high accuracy when classifying pathologies in the analyzed X-rays. In [8], the authors define a classification of pneumonia using pretrained convolutional neural network models. This Ensemble Learning method combines features extracted from three well-known CNN models (DenseNet169, MobileNetV2, and Vision Transformer) to achieve exceptional results with an accuracy of 93.91% and an F1-score of 93.88% on the test phase.

In [9], the authors address the increase in lung diseases by proposing a multiclass classification of 10 different lung pathologies using a refined CNN model. With the use of various pretrained networks, the proposed model, named LungNet22, achieves an accuracy of 98.89% through parameter optimization and the construction of a model derived from VGG16. Similarly, the study in [10] proposes a DL model to classify chest X-ray images into 14 different lung pathology conditions. Using transfer learning on pretrained neural networks such as DenseNet and ResNet, the model shows better accuracy than the competing network, highlighting the importance of data preprocessing to improve model performance.

In [11], the authors address the theme of the COVID-19 pandemic and its early identification using chest X-ray images. Through a model that combines a pretrained VGG19 network with three blocks of a convolutional neural network, the proposed approach achieves 96.48% accuracy, providing a reliable means to accelerate diagnosis and improve treatment efficiency. Similarly, in [12], an Ensemble model called PulDi-COVID is proposed for the diagnosis of nine lung diseases, including COVID-19. Using various pretrained neural networks and combining them through an Ensemble strategy, the approach offers remarkable results, achieving 99.70% accuracy, 98.68% precision, and 98.67% recall, thus highlighting its effectiveness in multipathological diagnosis. In [13], the analysis of post-COVID lung diseases is carried out using a combination of architecture to capture global features with Inception modules and a Transformer network to analyze local features. The use of an asymmetric loss function for multiclass classification demonstrates the superiority of the proposed model over other well-known architectures. The work in [14] focuses on globally relevant lung diseases such as pneumonia, COVID-19, tuberculosis, and pneumothorax. The proposed approach uses eight pretrained convolutional neural networks to automatically classify chest X-ray images. The best model, Densenet-201, achieves an accuracy of 97.2%, surpassing other state-of-the-art methods and demonstrating the potential for automation in the rapid diagnosis of lung diseases.

An approach for the detection of lung diseases through chest X-ray images, using an optimized Deep Convolutional Spiking Neural Network (DCSNN) with the Arithmetic Optimization Algorithm (AOA) and the Kaggle NIH dataset, is proposed in [15]. The method includes an image preprocessing phase, including an anisotropic diffusion filter and an enhancement scheme. The DCSNN, enhanced by AOA, achieves overall sensitivity of 31.87%, specificity of 26.88%, and recall of 28.14%, surpassing other methods in the literature, such as LDC-SVM-SMO and LDC-XGBoost-PSO.

In [16], the authors propose a neural network model called Lung-GANs for the classification of lung diseases from chest X-ray images. This approach uses a multilevel structure of generative adversarial networks (GANs) to learn interpretable representations of lung disease images from unlabeled data. The model eliminates the need for large labeled datasets, making it advantageous for new and complex lung diseases. During experiments, Lung-GANs outperforms the existing unsupervised models, achieving exceptional accuracy in the range of 94–99.5% on six extensive datasets of lung diseases. The strength of Lung-GANs lies in its ability to generalize without requiring a high amount of labeled data. The model is applicable to various classifications of lung diseases, such as TB vs. healthy, pneumonia vs. normal, COVID-19 vs. pneumonia, and COVID-19 vs. non-COVID, demonstrating superiority in terms of accuracy (up to 99.5%) and sensitivity compared to the existing methods.

In [17], the challenge of automatic lung segmentation in chest X-ray images is addressed. Through the use of a convolutional neural network with concatenation blocks and transpose layers, the proposed model achieves promising results with an accuracy of 97%, an IoU of 93%, and a Dice coefficient of 96%.

In summary, the current scientific studies in the literature show significant progress in the field of lung disease diagnosis through the use of sophisticated artificial intelligence and machine learning techniques. The implementation of these proposals offers new perspectives to improve the accuracy and efficiency of medical diagnosis, promoting rapid identification and timely treatment of lung diseases. In general, it can be concluded that the discussed progress has been driven by the development of

- More powerful DL architectures: new DL architectures, such as DenseNet, ResNet, Transformers, and Inception, have been developed to effectively capture complex features from X-ray images.
- Large X-ray datasets: the availability of large X-ray datasets, such as the NIH ChestX-ray14 dataset, has allowed the training of more accurate DL models.
- Collective learning: collective learning techniques, combining multiple DL models to improve performance, have been used to achieve the best results in lung disease detection.

The detection of lung diseases through DL techniques based on X-ray images has numerous potential applications, including

- Fast and accurate diagnosis: DL models can be implemented on mobile devices or in hospitals to provide rapid and accurate diagnosis of lung diseases.
- Early detection of lung cancer: DL models can be used to identify early signs of lung cancer, leading to early treatment and better outcomes for patients.
- Risk classification: DL models can be used to classify patients based on their risk of developing lung diseases, providing useful information for therapeutic decisionmaking.

In the current research landscape, studies focus exclusively on the application of DL techniques to chest images to optimize the classification of lung pathologies.

Regarding DT-based applications in the pulmonary field, the scientific literature has undergone little development in recent years. Some studies [18,19,20,21] propose DT-based systems to create virtual representations of the lungs to improve the diagnosis of diseases such as pneumonia and COVID-19.

For example, in [18], a Digital-Twin-based smart healthcare system combined with medical devices is proposed to collect information regarding the current health condition, configuration, and maintenance history of the device/machine/system. The system also analyzes medical images using a DL model to detect COVID-19. The system is based on the cascade recurrent convolution neural network (RCNN) architecture, achieving a mean average precision rate of 94%. In [19], a new system for telemedical simulation in remote lung cancer implementation is presented, combining DL, DTs, mixed reality, and medical IoT. The system aims to improve accuracy and immersion during remote surgical implementation. The system is based on a robust auxiliary classifier generative adversarial network (rAC-GAN) for patient-specific data analysis and prediction. The rAC-GAN model is trained on data from 90 lung cancer patients with pulmonary embolism (PE) and 1372 lung cancer control groups. The system achieved area under the curve (AUC) values of 92% and 93%, showing significant performance improvement in processing clinical data. The work conducted by the authors in [20] proposes a new Electrical Impedance Tomography (EIT) framework using DT models and DL to improve image quality and anti-noise performance. The proposed EIT framework incorporates DT models to generate a virtual dataset of EIT measurements and lung information. The framework also includes a DL-based image reconstruction network (IR-Net) to leverage labeled data and reconstruct conductivity distributions within the lungs during respiration. The IR-Net achieves superior image quality and anti-noise performance compared to the traditional EIT algorithms. Finally, in [21], an intelligent Internet of Medical Things (IoMT) platform for automatic pneumonia diagnosis in chest X-ray images is proposed. The platform utilizes DT technology to create a virtual representation of the lung based on real-time X-ray data. The article introduces an Enhanced Vision Transformer Model (EVTM) for analyzing chest X-ray images by using Vision Transformer technology to convert images into sequences for improved feature extraction. The model is trained on a dataset of chest X-ray images and outperforms baseline models, with higher precision, recall, accuracy, and F1-score values.

However, none of the abovementioned studies focus on developing a multilevel platform capable of acquiring, processing, storing, and sharing information for the prevention or diagnosis of pathologies related to abnormal values from multiple organs.

This study introduces an architecture based on DTs, incorporating multiple levels of abstraction. These levels encompass organ-based DTs and pathology-based DTs, with the integration of artificial intelligence models. In fact, the goal is not only to integrate the classification results obtained from neural network models related to lung images but to create a broader structure composed of various organ DTs, as in the case of the study on the heart in [3]. This architecture aims to accumulate multi-organ information to support doctors in the diagnosis and prevention of diseases (such as acute coronary syndrome), considering the interactions between different sub-pathologies related to each organ.

Research in the field of applying DT technology in the pulmonary context has made limited progress. Nevertheless, the implementation of DT technology in pulmonary settings offers several significant advantages in clinical contexts. Among these, the customization of treatment emerges as a promising perspective, allowing for the creation of highly detailed virtual models of a patient’s lungs. This personalized approach paves the way for targeted and specific therapies for individual lung conditions, enhancing the effectiveness of medical interventions. Another crucial advantage lies in the optimization of treatment. Through the use of DTs, physicians can virtually simulate the effects of various therapies and interventions, quickly identifying the most effective approach and reducing the risk of ineffective or invasive treatments. This not only contributes to improving outcomes for patients but also optimizes the use of healthcare resources.

Medical education is another area of significant benefit. The use of Digital Twins as advanced educational tools offers medical students and healthcare professionals the opportunity to explore the anatomy and dynamics of the lungs in detail within a virtual environment. This not only enhances theoretical understanding but also enables the development of practical skills through realistic simulations, better preparing healthcare professionals for complex clinical situations.

A central aspect that emerges strongly is cost reduction. The application of DTs in the pulmonary context can significantly contribute to reducing costs associated with misdiagnoses, ineffective treatments, and unnecessary surgical interventions. Through detailed simulations, physicians can make more informed decisions, thus reducing expenses related to diagnostic errors and improving the efficiency of healthcare services.

The main challenges that the architecture proposed in this study aims to address are the following:

- Continuous and personalized patient monitoring: use DTs to optimize real-time collection and processing of data from various medical devices, including wearable sensors, clinical records, and other applications. These data can be used to create a digital model of the patient, predict the risk of diseases, identify anomalies, and optimize treatment.
- Unified patient management platform: use a platform allowing monitoring of different organs and pathologies through the creation of organ DTs that, when working collaboratively, provide the medical world with a tool for a comprehensive view of the patient’s health condition, as well as treatment planning optimization capabilities.
- Optimization in the fusion and sharing clinical information: consult, through a single frontend, the entirety of patient medical information and create a shared virtual environment where doctors can actively collaborate to enhance patient care, share data, discuss clinical cases, and plan surgeries.
- Tool to improve procedures and reduce healthcare costs: use DTs in medical scenarios to simulate medical procedures in a safe virtual environment before performing them on a real patient, and receive feedback to improve organizational efficiency. These simulations can be a valuable tool to assess the critical points of the entire medical supply chain and optimize use of medical resources, such as medical devices and staff, to reduce healthcare costs.

The Table 1 outlines specific challenges and limitations present in various related studies, highlighting the gaps that require the development of the AI-powered DT framework, i.e. Lung-DT. This comparative assessment serves to justify the imperative need for Lung-DT by addressing and overcoming the shortcomings observed in current thoracic health monitoring methodologies.

## 3. Lung-DT

The proposed Lung-DT in this study represents an innovative approach in the field of medical DTs, allowing real-time acquisition of detailed data on the lung’s status. Through real-time data acquisition and continuous monitoring of specific inputs, the system can leverage this information for advanced diagnostic purposes using artificial intelligence algorithms. The essence of the Lung-DT lies in its ability to create a highly faithful virtual replica of the real lung. This replica goes beyond reflecting the current state; it can project potential future scenarios. The goal is not only to detect anomalies but also to anticipate them, enabling proactive intervention to improve lung health management.

Building on the architecture of the Heart Digital Twin (HDT) [3], the authors aim to extend the concept to create a DT of the lung. Similar to the approach adopted for the HDT, the creation of a Lung-DT involves various functional components in both the digital and real-world domains. This extension enriches the platform, requiring the implementation of scalable, dynamic, and resilient systems, along with a modular approach. In addition to scalability, dynamism, and resilience, robustness is a key consideration in the development of Lung-DT. This comprehensive approach aims to create a Lung-DT platform that not only adapts to changing demands but also remains steadfast and reliable in various circumstances.

Similar to the creation of an HDT, a microservices approach was adopted for the design and implementation of Lung-DT’s various functional blocks. This architectural decision allows for the creation of modular and independently deployable services, contributing to the overall flexibility, maintainability, and robustness of the system.

Microservices were implemented using containerization technologies, specifically Docker, to encapsulate each functional block into separate lightweight containers. These containers provide isolation and portability, ensuring consistent behavior across various environments. The emphasis on robustness means that the Lung-DT can maintain its functionality and performance in case of unexpected disruptions or challenges. Kubernetes was employed as the orchestration tool to manage and scale these Docker containers efficiently. This robust orchestration ensures seamless coordination of microservices, contributing to the overall robustness of the Lung-DT platform.

All the functional blocks of the Lung-DT were implemented as Pods within the Kubernetes cluster, each Pod containing various Docker containers representing individual microservices. This design facilitates easy management, scaling, and maintenance of various components within the Lung-DT ecosystem, while concurrently enhancing the robustness of the overall system.

### 3.1. Lung-DT Architecture

Lung architecture characterizes the DT based on the specific context. In lung pathology scenarios, input data may be of various types, including numerical data and X-ray images. Unlike the Heart-DT, the Lung-DT must be able to acquire images. Therefore, in addition to the two connectors, there are two distinct Acquisition blocks to process raw data from the real world.

The two present connectors are dedicated to acquiring the SpO2 value from a smartwatch and data from lung X-rays. In addition to the connectors, the data Acquisition block related to the X-ray connector performs resize and categorization operations on the images before storing them in the Storage block. Subsequently, the Storage block is responsible for storing preprocessed data for future use.

The Agent block initiates different decisionmaking processes based on the input data in the storage and programmatically triggers data analysis. Depending on the nature of the analysis, the agent will activate the AI Process block, which processes images or saturation data.

The AI Process block can download various models from the AI Service block, i.e. the reference model for making inferences based on the required analysis. The following chapters will describe the methodology and results of the inference process in which the Lung-DT is required to process lung images.

Finally, once the Agent block obtains the result of processing, it will assess the necessary actions, such as sharing information with other DTs or implementing actions in the real world through the implementation block. Similar to the heart case, the Policy block holds all policies and thresholds to prevent actions that may be harmful to the organ itself.

**Figure 1:**
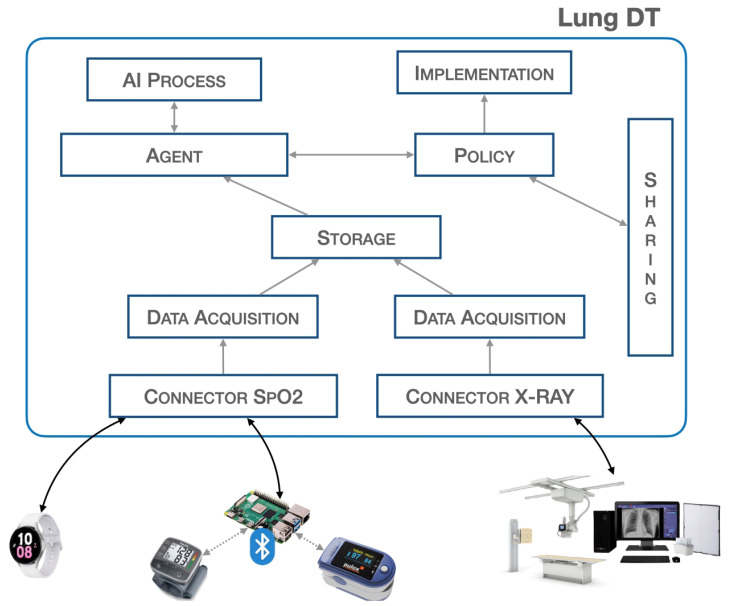
Lung-DT architecture.

As shown in Figure 1, the implementation provided in this study on the Lung-DT involves two distinct connectors, both designed to interface directly with the physical world for data acquisition. The first connector is used to acquire SpO2 values from wearable medical devices, such as a smartwatch, while the second is dedicated to acquiring X-ray images from the real world.

### 3.2. Lung-DT Workflow

**Figure 2:**
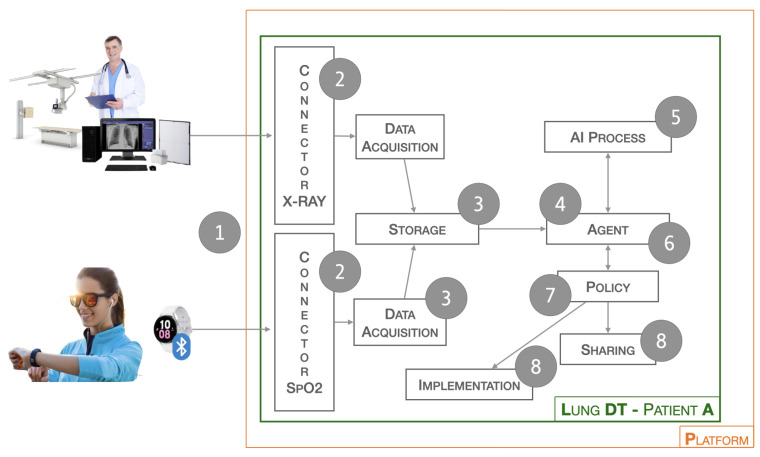
Workflow of Lung-DT.

The workflow related to the Lung-DT is illustrated below. Data from the real world are acquired through a single endpoint that IoT devices and various systems can connect to. The platform exposes various services, as shown in Figure 2, with two connectors (1) dedicated to data acquisition. For saturation data, they are sent via REST API or MQTT, while, for X-ray images, there is an FTP repository where files can be uploaded. It is essential to emphasize that medical personnel uploading the clinical report will only have access to the Lung-DT repository specific to the individual patient.

The next phase (2) involves reading the data and performing cleaning and normalization operations. In this process, operations such as the removal of non-useful metadata, uniformity of format, and file type are carried out. The Storage block (3) is responsible for storing the raw and cleaned data for future use.

The Agent block (4), through cyclical checks or external interactions, initiates the validation and inference process for previously acquired data. In particular, if a new image has been received, it will contact the AI Process block (5) to start the validation process. In the case of an X-ray image, the AI Process block will contact the AI Long Instance X-ray block to check for the presence of a new updated model, which will be used for inference.

Once completed, the result is returned to the Agent block (6) to assess future actions. Specifically, based on the thresholds set in the Policy block (7), certain actions will or will not be taken. These actions may include physical world actions through the Implementation block or sharing with other DTs through the Sharing block (8).

The Implementation block (8) is utilized in the context of this study to implement and establish alert and notification systems for medical specialists. This enables them to promptly consider the possibility of activating remote administration systems, scheduling specific medical exams, or triggering immediate emergency services. The objective is to develop a modular and dynamic architecture capable of adapting to various application scenarios and, above all, facilitating seamless integration of new functionalities without compromising the integrity of the entire system, as previously proposed by the authors in [3].

### 3.3. Healthcare DT Platform

**Figure 3:**
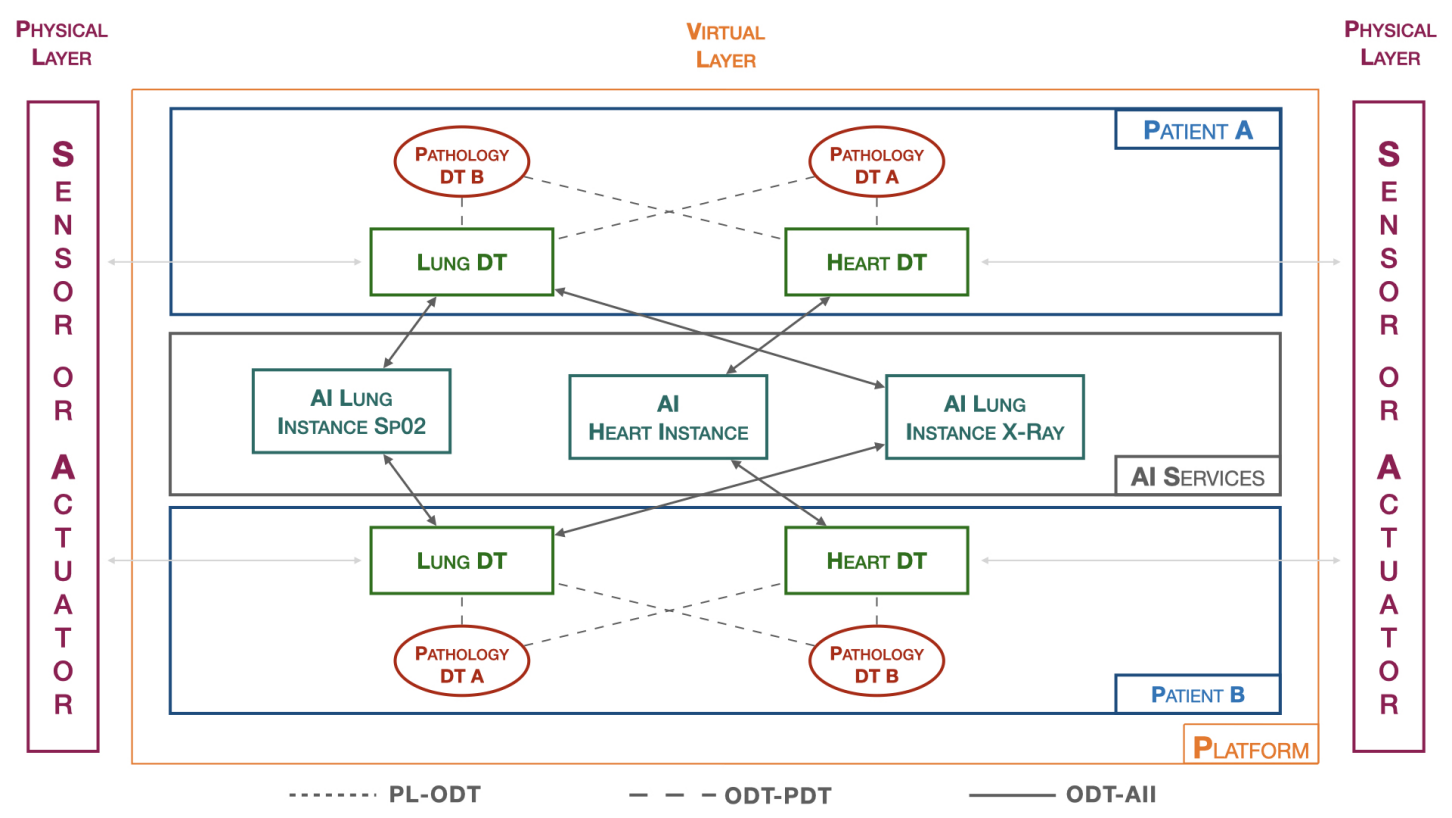
Platform architecture.

The platform introduced by the authors [3] has been characterized for a specific application related to the lung, as illustrated in Figure 3. In the patient-dedicated section, there are DTs related to both heart and lung, while two generic DTs for pathologies are exemplified. The AI Services section includes three instances, one focused on the cardiac context (AI Heart Instance), while the other two are dedicated to the pulmonary domain. Specifically, there is the AI Lung SpO2 Instance, related to blood saturation data, and the AI Lung X-ray Instance, which handles the processing of lung images. All instances are independent of each other, allowing updates and changes without affecting the behavior of the entire platform. This flexibility enables adapting the platform to specific needs, facilitating the seamless integration of new elements to enhance the overall capabilities of the system. The modular structure of the platform provides the opportunity to customize and expand functionalities based on the evolving requirements of the application.

## 4. Testbed Setup of Lung-DT

This section will outline the steps that led to the creation of the neural network model integrated into Lung-DT, capable of classifying chest X-ray images into five classes of lung diseases. Specifically, we will describe two public datasets used in this study, the preprocessing phase of the images before their input into the network, the convolutional neural network model used with its respective stages (training, validation, and testing), and, finally, the results obtained during the training and testing phases of the neural network.

### 4.1. Datasets

In this section, we will illustrate the dataset used to train, validate, and test the neural network, described in Section 4.2, for classifying five different classes of lung pathologies, including the “normal” class. Specifically, to have five different pathology classes and a significant number of lung images (from different individuals with different genders and ages) for training and validating the network, two open datasets on Kaggle were used: the “Multiclass Chest X-ray Disease Dataset” [22] and the “LungsDiseaseDataset(4types)” [23].

The first dataset [22] contains chest X-ray images in various formats (PNG and JPEG) and includes four classes of lung diseases (pneumonia, COVID-19, tuberculosis, and lung_opacity) and one “normal” class. The number of images in each class is as follows: 1583 for the “normal” class, 4273 for “Pneumonia” class, 6011 for “lung_opacity” class, 4192 for “covid” class, and 703 for “tuberculosis” class.

The second dataset [23] is similar to the first, containing chest X-ray images in JPEG format. This dataset was created by integrating other publicly available datasets, excluding the first one mentioned. It presents four classes of lung diseases (bacterial and viral pneumonia, coronavirus disease, and tuberculosis) and a “normal” class. To ensure a significant amount of data, the data augmentation factor of 6 was applied, resulting in just over 10,000 images distributed as follows: 2013 for the “normal” class, 2009 for “bacterial pneumonia”, 2008 for “viral pneumonia”, 2031 for “corona virus disease”, and 2075 for “tuberculosis”.

These two datasets were chosen to create a unified dataset for training and validating the YOLOv8 neural network. In particular, for each class, the images were combined, ensuring there were no duplicates, and the format of all images was standardized to PNG and resized at 224 × 224.

**Figure 4:**
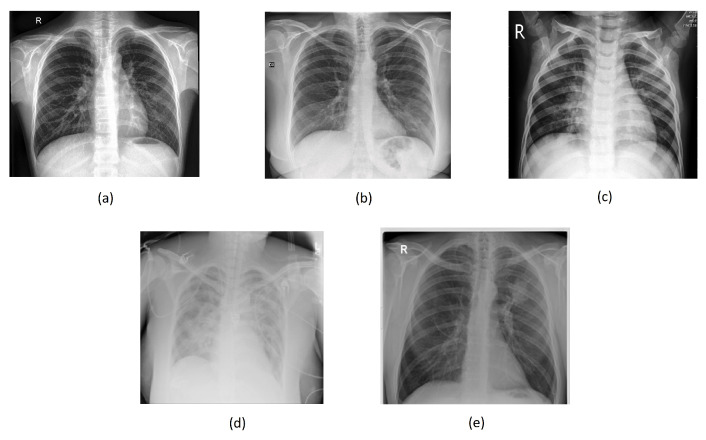
Chest images for (**a**) “normal”, (**b**) “covid”, (**c**) “pneumonia”, (**d**) “lung_opacity”, and (**e**) “tuberculosis” classes.

Figure 4a–e below illustrate the “normal”, “covid”, “lung_opacity”, “pneumonia”, and “tuberculosis” classes.

The unified dataset contains a total of 23,442 images and was subsequently randomly divided into Training Set and Validation Set according to the following proportions: 70% and 30%. Therefore, the number of elements within each class for the Training Set is as follows:

- “normal”: 1481 images;
- “covid”: 3997 images;
- “pneumonia”: 5348 images;
- “lung_ppacity”: 4207 images;
- “tuberculosis”: 1330 images.

The number of elements within each class for the Validation Set is as follows:

- “normal”: 635 images;
- “covid”: 1697 images;
- “pneumonia”: 2291 images;
- “lung_opacity”: 1803 images;
- “tuberculosis”: 653 images.

Other public datasets include “Multi_Classe_Chest X-ray_DATASET(VERSION2)” [24], “Tuberculosis Chest X-rays (Shenzhen)” [25], “Chest X-rays tuberculosis from India” [26], and “Balanced Augmented Covid CXR Dataset” [27] (also available on Kaggle). The datasets were chosen to create a single testing dataset containing images that are entirely unknown to the network for each of the five classes of lung pathologies. Before describing the technique adopted to merge the datasets, a brief description of each is provided below.

The dataset [24] includes four main classes: “covid”, “normal”, “pneumonia”, and “lung cancer”. The images in the dataset have various formats, including PNG and JPEG. Specifically, the dataset consists of 106 images for the “covid” class, 132 images for the “normal” class, 103 images for “pneumonia”, and 49 images for “lung cancer”.

The datasets [25] have been collected and provided by Shenzhen No.3 Hospital in Shenzhen, Guangdong Providence, China. The images are in the PNG format. There are 326 normal and 336 tuberculosis chest X-rays, respectively. The dataset also includes consensus annotations for a subset (N = 68) from two radiologists for 1024 × 1024 resized images and radiology readings.

The datasets [26] come from Jaypee University of Information Technology (India). There are 77 normal and 77 tuberculosis chest X-rays, respectively. The images are in the JPEG format.

The datasets [27] include four main classes: “covid”, “normal”, “viral pneumonia”, and “lung_opacity”. The images are in the PNG format. Specifically, the dataset consists of 7845 images for the “covid” class, 8214 images for the “normal” class, 5365 images for “viral pneumonia”, and 7674 images for “lung_opacity”.

The testing dataset was created following the criteria below: the “covid”, “normal”, and “pneumonia” classes were taken from the dataset [24]; the “lung_opacity” class was extracted from the dataset [27], considering only 5% of the images (randomly selected); the “tuberculosis” class was formed by merging the two “tuberculosis” classes present in [25,26]. The number of images in each class for the resulting testing dataset are as follows:

- “normal”: 132 images;
- “covid”: 106 images;
- “pneumonia”: 103 images;
- “lung_opacity”: 383 images;
- “tuberculosis”: 423 images.

Also in this case, as for the training and validation datasets, the format of all images was standardized to PNG and resized at 224 × 224.

It is important to underline that these datasets are made up of lung images belonging to both men and women; very often, the age range and ethnicity are not indicated. Overall, we used a very large dataset that manages to include a wide range of examples.

### 4.2. Convolutional Neural Network: YOLOv8

YOLOv8 [28] is the latest model in the YOLO (You Only Look Once) series, designed for object detection, image classification, and instance segmentation. Developed by Ultralytics, also known for the YOLOv5 model, YOLOv8 introduces numerous architectural changes and improvements in the developer experience compared to YOLOv5.

The YOLO series of models gained prominence in the computer vision community due to its remarkable accuracy and compact model size.

Noteworthy studies [29,30] in the literature have specifically leveraged YOLOv8 for image classification tasks, demonstrating superior performance compared to conventional approaches. YOLO was initially implemented in C in versions 1-4, using a custom DL framework called Darknet. YOLOv8 emerged as part of the YOLOv5 development process when Glenn Jocher of Ultralytics began contributing to the YOLOv3 repository in PyTorch.

YOLOv8 is characterized by several features that make it appealing for computer vision projects:

- High Accuracy: YOLOv8 has demonstrated high accuracy measured through COCO and Roboflow 100 metrics.
- Developer Convenience: the model offers many features for developer convenience, including an easy-to-use command-line interface (CLI) and a well-structured Python package.
- Large Community: YOLO has a large community, and the YOLOv8 community is growing. This means there are many online resources and experts in computer vision who can provide support and guidance.

YOLOv8 attains impressive accuracy on COCO. Specifically, the YOLOv8m model, which is of medium size, achieves a mAP of 50.2% when assessed on COCO. In comparison to YOLOv5, YOLOv8 demonstrates significant superiority when evaluated on Roboflow 100, a dataset designed explicitly to evaluate model performance across diverse task domains. Additional information on this is elaborated in our subsequent performance analysis within the article.

Moreover, the developer-friendly features in YOLOv8 are significant. Unlike other models where tasks are distributed across many different Python files that need to be run, YOLOv8 comes with a CLI that makes model training more intuitive. This is in addition to a Python package that provides smoother coding experience compared to previous models.

Despite the absence of an official paper, some features of the YOLOv8 architecture have been analyzed by Ultralytics. Some key updates include

- Anchor-Free Detection: YOLOv8 adopts an anchor-free model, predicting the object’s center directly rather than the offset from a known anchored box.
- New Convolutions: changes have been made to the model’s structure, including the replacement of 6 × 6 convolutions with 3 × 3 convolutions and modifications to the main building blocks.
- Mosaic Augmentation Closure: YOLOv8 uses mosaic augmentation during online training but disables this technique in the last ten epochs to improve performance.

Therefore, the network is composed of four main components: backbone, neck, head, and loss.

- Backbone is the part of the network responsible for extracting visual features from the input images. In our implementation, we used the PPM (Path Aggregation Network) backbone, which is a variant of the Darknet-53 backbone. PPM combines features from different scales using a max-pooling pyramid module, which allows the network to learn visual representations at multiple resolutions, improving the accuracy of classification;
- Neck is an intermediate component that refines the features extracted from the backbone and prepares them for the classification process. In our implementation, we used the CSPHead (Cross Stage Partial Network) neck, which combines concatenation and fusion to generate more informative features. CSPHead is able to capture both global and local relationships in images, improving the accuracy of classification;
- Head is the final part of the network responsible for classifying the images;
- Loss is a function that measures the accuracy of the network during training.

**Figure 5:**
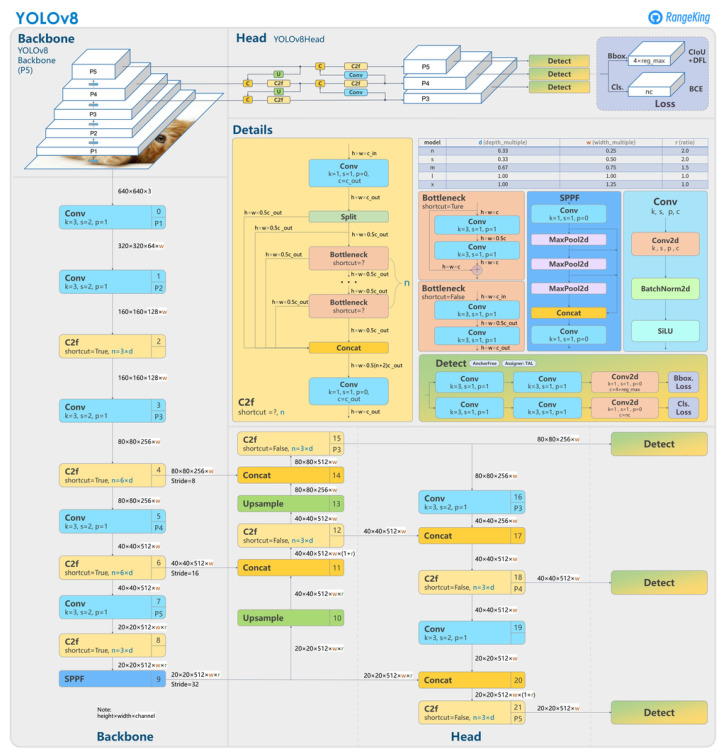
YOLOv8 architecture.

The architecture of the YOLOv8 network is defined in Figure 5.

The training parameters are the values that govern the learning process of the neural network. In our implementation, we used the following training parameters: learning rate equal to 0.001, batch size equal to 32, and number of epochs equalling 100. The optimization techniques are algorithms used to minimize the loss during training. In our implementation, we used the AdamW (Adaptive Moment Estimation with Weight Decay) optimization method. AdamW is a variant of the Adam algorithm that incorporates an additional penalty for large gradient values, improving training stability. Fine-tuning is an advanced training technique that allows a pretrained network to be adapted to a new domain or task. In our implementation, we applied a fine-tuning process using a dataset of chest X-rays containing images of patients with different medical conditions. In order to obtain a neural network model capable of classifying lung pathologies into five classes, this network was trained with fine-tuning, setting the initial weights corresponding to those used by the author on the model trained on COCO. Subsequently, 100 training epochs were conducted, resulting in an average accuracy of 96.8%.

### 4.3. Results

**Figure 6:**
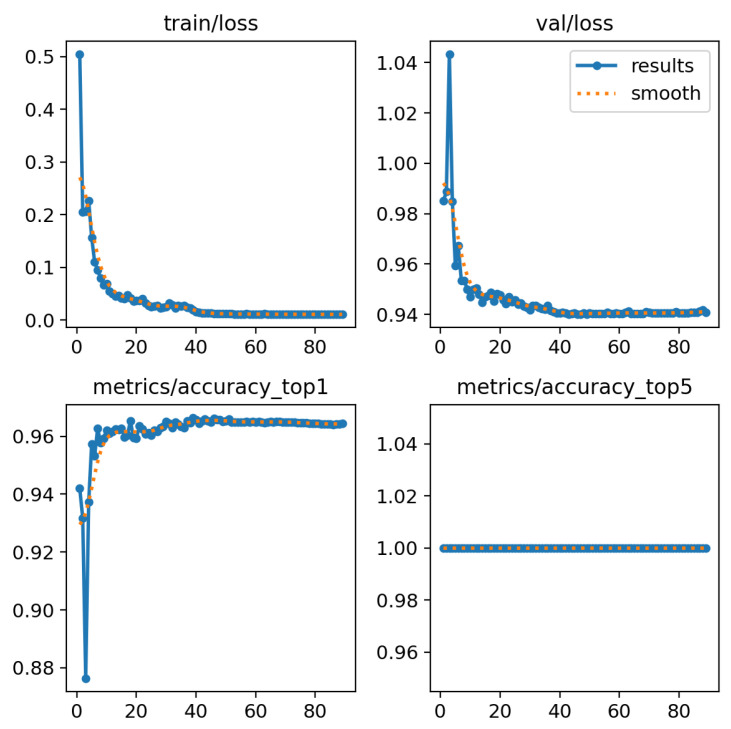
Results of YOLOv8 using validation set.

Following the detailed description of the neural network architecture and training methodology outlined in the previous section, we present the outcomes and performance metrics of our chest X-ray classification model. To assess the model’s learning dynamics over training epochs, we visualize the accuracy and loss trends on both the training and validation sets across 100 epochs in Figure 6. These graphs offer insights into the convergence and generalization capabilities of our neural network.

**Figure 7:**
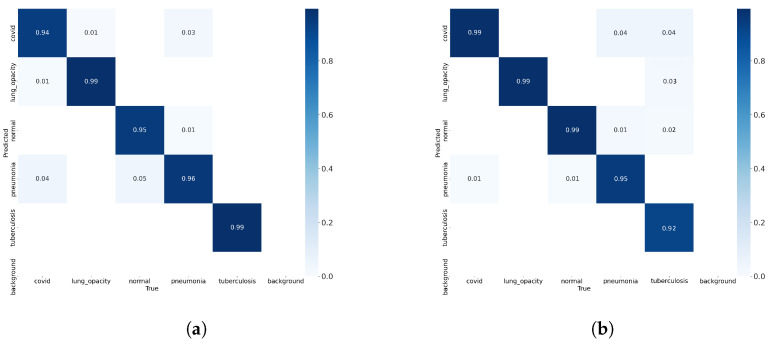
Confusion matrix using (**a**) validation test, (**b**) testing test.

In Figure 7a, a comprehensive view of the model’s performance on the validation set is provided through a confusion matrix. Notably, the matrix indicates an impressive average accuracy of 96.6%, reflecting the model’s proficiency in classifying chest X-rays within a familiar data distribution. Subsequently, the model was subjected to an extensive testing phase using a dataset completely unknown to the network (not belonging to either the training set or the validation set). The results, showcased in Figure 7b, depict a confusion matrix for the testing dataset, revealing an exceptional accuracy of 96.8%.

**Figure 8:**
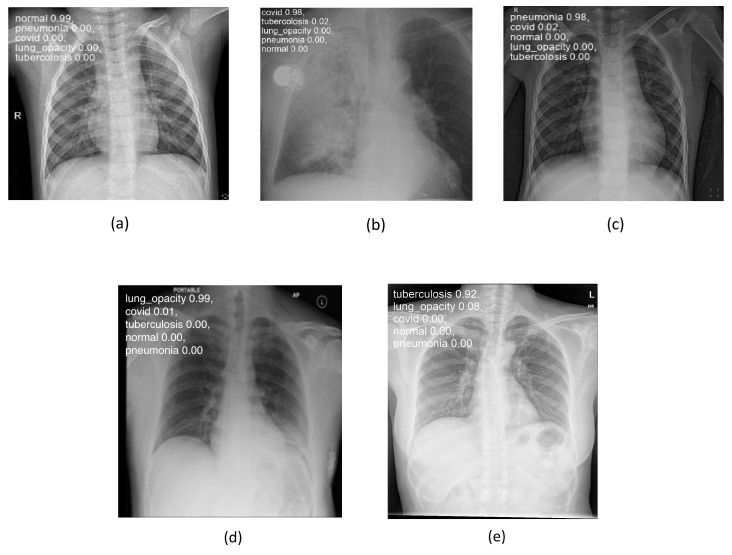
Lung disease classification for (**a**) “normal”, (**b**) “covid”, (**c**) “pneumonia”, (**d**) “lung_opacity”, and (**e**) “tuberculosis” classes using testing set.

Figure 8 shows some examples of lung images, each corresponding to its respective pathology, with the classification percentage result for all classes. As can be observed, the model performs very well even on images it has never seen before, achieving a rate of accuracy ranging from 92 to 99%.

In addition to accuracy, the evaluation strategy incorporates key performance metrics, including precision, recall, and F1-score, to provide a thorough assessment of the system’s capabilities in handling various aspects of classification. These metrics contribute to a comprehensive understanding of the model’s robustness and efficacy in real-world applications. The precision metric measures the accuracy of positive predictions among all instances predicted as positive, expressed mathematically in (1).

(1) $$\mathrm{Precision}=\frac{\mathrm{True}\;\mathrm{Positives}}{\mathrm{True}\;\mathrm{Positives}\;+\;\mathrm{False}\;\mathrm{Positives}}$$

Recall, also known as sensitivity or true positive rate, gauges the model’s ability to correctly identify all relevant instances, and it is calculated in (2).

(2) $$\mathrm{Recall}=\frac{\mathrm{True}\;\mathrm{Positives}}{\mathrm{True}\;\mathrm{Positives}\;+\;\mathrm{False}\;\mathrm{Negatives}}$$

The F1-score, expressed mathematically in (3), defined as the harmonic mean of precision and recall, strikes a balance between the two and is particularly useful in scenarios with imbalanced class distributions.

(3) $$F1-\mathrm{score}=\frac{2\;\cdot \;\mathrm{Precision}\;\cdot \;\mathrm{Recall}}{\mathrm{Precision}\;+\;\mathrm{Recall}}$$

These metrics collectively offer a robust evaluation framework, enabling us to assess the model’s performance across various aspects, from precision-focused to recall-focused scenarios. Additionally, accuracy must be considered, i.e. the ratio of correctly predicted instances to the total number of instances, expressed in (4) to provide a holistic view of overall model performance.

(4) $$\mathrm{Accuracy}=\frac{\mathrm{True}\;\mathrm{Positives}\;+\;\mathrm{True}\;\mathrm{Negatives}}{\mathrm{True}\;\mathrm{Positives}\;+\;\mathrm{True}\;\mathrm{Negatives}\;+\;\mathrm{False}\;\mathrm{Positive}\;+\;\mathrm{False}\;\mathrm{Negative}}$$

Based on the metrics mentioned above, the model delivers excellent performance on the testing dataset. The model’s precision is approximately 92%, the recall is 97.0%, and the obtained F1-score is 94%. Finally, the model accuracy, as previously mentioned, is about 96.8%.

## 5. Discussion

This section provides a comprehensive discussion and comparison of the current state-of-the-art research in the field and the Lung-DT proposed in this paper.

Table 2 presents a comparative analysis between our study and the existing literature regarding the application of DL techniques for classifying X-ray images to detect specific pulmonary pathologies.

The table specifically contrasts the employed technologies, the identified classes, and the performance metrics, particularly focusing on accuracy.

Considering the references reported in Table 2, the present study is characterized by two fundamental innovations. The first relates to the improvement in the classification process, while the second focuses on the application of the DT paradigm.

In terms of the classification, the dataset for training and validation of the model was created by merging two distinct public datasets, whereas the testing dataset was formed from four public datasets containing images completely unknown to the network. This strategic choice aims to ensure a level of completeness and robustness in managing data diversity. To confirm this, high performance rates were achieved using the aforementioned testing dataset, which is entirely unrelated to the training dataset—a crucial aspect introduced in this study.

The obtained results indicate that accuracy, F1-score, precision, and recall are comparable or superior to the average rates found in the literature. It is worth noting that the present study stands out as five classes in the classification process are considered, whereas studies available in the literature typically distinguish between two and four classes of pulmonary pathologies. These results suggest the effectiveness of the model in the classification task compared to the traditional approaches.

In terms of the use of the DT paradigm, the Lung-DT characterized by pulmonary X-ray images was implemented. The incorporation of a Lung-DT within the platform allows for extending the monitoring of patients’ health through collaboration between DTs of different organs.

## 6. Ethical Aspects and Hypothetical Example

The integration of AI algorithms in the medical field raises not only technical concerns but also crucial ethical issues. Among the identified problems, the following stand out:

- Equity: In the medical context, equity becomes a primary concern as AI algorithms, trained on demographically unbalanced data, may introduce biases into medical decisions. For example, if an algorithm is predominantly trained on data from patients of a specific ethnicity, it may exhibit lower accuracy in diagnosing diseases in individuals of other ethnicities, leading to disparities in healthcare and research outcomes.
- Responsibility: Responsibility emerges as a crucial point in the presence of automated medical decisions. Identifying the responsible party in the case of negative outcomes is complex and requires clarity. The chain of responsibility may involve the physician using the algorithm, the programmer who developed it, or the manufacturing company. Defining norms and standards for responsibility is essential to ensure ethical use of algorithms in the medical context.
- Transparency: Transparency plays a fundamental role so that both physicians and patients can understand how AI algorithms operate. The complexity of many algorithms can make interpreting decisions challenging, undermining trust and limiting the assessment of accuracy and fairness in recommendations. Making algorithms more accessible and understandable is therefore crucial to promote trust and ensure ethical use of data in the medical domain.

To address these ethical issues, specific approaches must be adopted; e.g., to mitigate equity concerns, it is essential to use diverse and inclusive training datasets, incorporating a wide range of demographic data. Additionally, developing algorithms capable of identifying and mitigating biases during the decisionmaking process is a key strategy.

Addressing responsibility requires clear definition of norms and standards for each phase of the algorithm’s life cycle, along with the implementation of regular control and review mechanisms.

Improved transparency may be achieved by making algorithms more understandable through the use of interpretable models and providing continuous training to medical professionals and patients.

While these issues are still under study, the commitment of the medical and scientific sectors aims to ensure safe, fair, and ethical use of AI algorithms, fostering trust between medical professionals and patients, as well as enhancing the overall quality of healthcare.

The impact of AI algorithms in medical settings goes beyond technical and ethical concerns. False diagnoses generated by these algorithms can have profound implications for patients. On the one hand, false positives may lead to unnecessary anxiety and interventions, subjecting patients to medical procedures that are not essential. On the other hand, missed diagnoses can result in severe health consequences for patients.

The importance of ongoing scientific research becomes evident in this context. Research plays a crucial role in minimizing the occurrence of false diagnoses, ensuring the accuracy and reliability of AI algorithms in medical applications. Continuous scientific exploration and improvement contribute to refining these algorithms, making them more effective, and reducing the risk of both false positives and false negatives.

In a hypothetical scenario, let us envision a patient incorporating a smartwatch integrated with the Lung-DT platform to monitor their health. This device can capture critical parameters such as respiratory rate, oxygen saturation levels, and physical activity.

The smartwatch is configured to securely transmit real-time data to the Lung-DT platform. Additionally, the Lung-DT platform collects the patient’s radiographs, integrating them into the system for a comprehensive assessment of lung health by combining physiological data with diagnostic visual information.

The outcomes of this analysis empower the system to identify and report anomalies or potential health issues identified in the integrated analyses. These notifications can be sent to both the user’s smartwatch and healthcare personnel, facilitating timely intervention based on a detailed assessment of the situation. This approach offers several advantages. Firstly, it enables real-time monitoring of the patient’s health condition via the smartwatch, significantly reducing the need for frequent hospital visits. It not only saves time for the patient but also alleviates the burden on hospitals, decreasing queues and waiting times. Moreover, continuous data analysis through the Lung-DT platform allows for an accurate evaluation of lung health without the need for expensive traditional diagnostic procedures. It reduces costs associated with such procedures and helps prevent overcrowding the healthcare system, enabling more efficient resource management.

Lastly, the immediate notifications generated by the platform in the case of anomalies provide a crucial advantage, allowing a prompt response to data variations. This approach not only enhances the effectiveness of treatments but also reduces the necessity for more intensive and costly medical interventions that may arise from situations not detected in real time.

## 7. Conclusions

The present study represents a significant contribution in the field of lung disease classification through the innovative use of DT technology. The results obtained highlight the effectiveness of the proposed approach, which distinguishes itself in several key aspects compared to the state of the art.

The conducted research focuses on the implementation of a practical case study, providing a proof of concept for a Lung-DT. This implementation is characterized by a microservices architecture, integrating the artificial intelligence component that operates on heterogeneous input signals. Specifically, the proposed Lung-DT architecture receives data from chest X-ray images and blood oxygen saturation, allowing for a more comprehensive and detailed evaluation.

The novelty of our proposal also arises from the extension of the second-level architecture previously presented in [3], incorporating DTs related to other organs, such as the lung in addition to the heart. This multilevel approach enables the identification and classification of a wide range of pathologies, leveraging information from various body systems.

In the context of training the neural network model based on YOLOv8, the integration of two open datasets has proven its effectiveness, achieving average accuracy on the validation dataset equal to 96.6% in the classification of lung pathologies into five classes. Furthermore, tests on a completely unknown dataset confirmed the high robustness of the proposed model, with an average accuracy of 96.8%, precision of 92%, recall of 97%, and F1-score of 94%.

It must be noted that the performance achieved in the present study often surpasses the rates found in the literature, suggesting a significant advancement in the application of DTs to the classification of lung pathologies. The proposed multilevel architecture, which incorporates models related to other organs such as the heart, offers an innovative approach to the diagnosis and classification of pathologies related to multi-organ issues. This work substantially contributes to the progress of computational medicine and technology-assisted diagnostics using DT technology.

In the future, expanding the Lung-DT with additional data, such as spirometry data or other information from various sources that can provide more detailed characteristics of the lung, is certainly crucial. The integration of such supplementary data will enrich the understanding of the pulmonary system, allowing the DT to provide a more comprehensive and detailed representation. Moreover, it is essential to explore and develop new artificial intelligence algorithms aimed at efficiently processing all acquired data. This ambitious approach aims to refine Lung-DT, transforming it into a highly effective tool in the medical domain. The implementation of advanced algorithms will contribute to a more precise interpretation of the collected data, thus improving the ability to diagnose and monitor lung conditions promptly and accurately.

## Figures and Tables

**Table 1:** A comparative analysis of the existing thoracic health monitoring methods.

Ref.	Main Technology	Primary Application	Architecture/Model	Significant Results	Key Points
[18]	Digital Twins, Deep Learning	COVID-19 detection through X-ray	Cascade Recurrent Convolution Neural Network (RCNN)	Mean Average Precision Rate of 0.94 in COVID-19 detection	Use of Digital Twins to enhance medical device management
[19]	Internet of Medical Things (IoMT), Digital Twins, 5G, GAN	Telemedical simulation for lung cancer	Robust intelligent network based on auxiliary classifier GAN (RAC-GAN)	Remote implementation of lung cancer surgery	Remote implementation of surgery for lung cancer
[20]	Electrical Impedance Tomography (EIT), Digital Twins, Deep Learning	Lung monitoring with EIT and deep learning	3D Digital Twin Lung Model, Image Reconstruction Network (IR-Net)	Improved accuracy and anti-noise in EIT compared to traditional methods	Utilization of a dynamic digital twin model for EIT
[21]	IoMT, Digital Twins, Enhanced Vision Transformer Model (EVTM), Deep Learning	Automatic pneumonia diagnosis	Enhanced Vision Transformer Model (EVTM)	High accuracy in pneumonia diagnosis	Establishment of an intelligent IoMT platform for pneumonia diagnosis
Our method	IoT, Digital Twins, YOLOv8 Neural Network, Deep Learning	Classification of lung diseases through X-ray	YOLOv8 Neural Network	Average accuracy of 96.8% on X-ray datasets	Real-time monitoring and automatic diagnosis of lung diseases

**Table 2:** Results comparison with the state of the art.

Ref.	Technology Used	Classes	F1-Score	Recall	Precision	Accuracy
[5]	Adaptive histogram equalization (CLAHE), (SVM), VGG19, and CNN networks	covid-lung_opacity-normal-viral pneumonia	-	0.89	0.91	0.91
[6]	CNN-SqueezeNet	normal-pneumonia	-	0.92	-	0.94
[8]	CNN, DenseNet169, MobileNetV2,Vision Transformer	normal-pneumonia	0.94	0.93	0.94	0.94
[7]	Multi-objective genetic algorithm, neural networks with fuzzy logic	norma-pneumonia	-	0.79–0.98	-	0.79–0.98
[18]	RCNN, DT	covid	-	-	0.94	0.94
[21]	Enhanced Vision Transformer Model, DT	normal-covid-pneumonia	0.91	0.99	0.90	0.88
Our method	CNN-YOLOv8, IoT, DT	normal-covid-pneumonia-lung_opacity-tuberculosis	0.94	0.97	0.92	0.97

## Data Availability

The datasets used for this study can be obtained from the sources mentioned in References [22,23,24,25,26,27].

## Abbreviations

The following abbreviations are used in this manuscript:

AI	Artificial Intelligence
CNN	Convolutional Neural Network
DL	Deep Learning
DT	Digital Twin
FTP	File Tranfer Protocol
GAN	Generative Adversarial Network
HDT	Heart Digital Twin
IoT	Internet of Things
MQTT	Message-Queuing Telemetry Transport
RNN	Recurrent Neural Network
YOLO	You Only Look Once

## References

[1] Wang X., Peng Y., Lu L., Lu Z., Bagheri M., Summers R.M. ChestX-ray8: Hospital-Scale Chest X-ray Database and Benchmarks on Weakly-Supervised Classification and Localization of Common Thorax Diseases. Proceedings of the 2017 IEEE Conference on Computer Vision and Pattern Recognition (CVPR).

[2] Kermany D.S., Goldbaum M., Cai W., Valentim C.C., Liang H., Baxter S.L., McKeown A., Yang G., Wu X., Yan F. (2018). Identifying Medical Diagnoses and Treatable Diseases by Image-Based Deep Learning. Cell.

[3] Avanzato R., Beritelli F., Lombardo A., Ricci C. (2023). Heart DT: Monitoring and Preventing Cardiac Pathologies Using AI and IoT Sensors. Future Internet.

[4] Gazda M., Plavka J., Gazda J., Drotar P. (2021). Self-supervised deep convolutional neural network for chest X-ray classification. IEEE Access.

[5] Hussein F., Mughaid A., AlZu’bi S., El-Salhi S.M., Abuhaija B., Abualigah L., Gandomi A.H. (2022). Hybrid clahe-cnn deep neural networks for classifying lung diseases from X-ray acquisitions. Electronics.

[6] Avanzato R., Beritelli F. Thorax Disease Classification based on the Convolutional Network SqueezeNet. Proceedings of the 12th IEEE International Conference on Intelligent Data Acquisition and Advanced Computing Systems: Technology and Applications.

[7] Varela-Santos S., Melin P. (2021). A new modular neural network approach with fuzzy response integration for lung disease classification based on multiple objective feature optimization in chest X-ray images. Expert Syst. Appl..

[8] Mabrouk A., Díaz Redondo R.P., Dahou A., Abd Elaziz M., Kayed M. (2022). Pneumonia detection on chest X-ray images using ensemble of deep convolutional neural networks. Appl. Sci..

[9] Shamrat F.J.M., Azam S., Karim A., Islam R., Tasnim Z., Ghosh P., De Boer F. (2022). LungNet22: A fine-tuned model for multiclass classification and prediction of lung disease using X-ray images. J. Pers. Med..

[10] Fan R., Bu S. (2022). Transfer-learning-based approach for the diagnosis of lung diseases from chest X-ray images. Entropy.

[11] Alshmrani G.M.M., Ni Q., Jiang R., Pervaiz H., Elshennawy N.M. (2023). A deep learning architecture for multi-class lung diseases classification using chest X-ray (CXR) images. Alex. Eng. J..

[12] Bhosale Y.H., Patnaik K.S. (2023). PulDi-COVID: Chronic obstructive pulmonary (lung) diseases with COVID-19 classification using ensemble deep convolutional neural network from chest X-ray images to minimize severity and mortality rates. Biomed. Signal Process. Control.

[13] Mezina A., Burget R. (2024). Detection of post-COVID-19-related pulmonary diseases in X-ray images using Vision Transformer-based neural network. Biomed. Signal Process. Control.

[14] Karaddi S.H., Sharma L.D. (2023). Automated multi-class classification of lung diseases from CXR-images using pre-trained convolutional neural networks. Expert Syst. Appl..

[15] Rajagopal R., Karthick R., Meenalochini P., Kalaichelvi T. (2023). Deep Convolutional Spiking Neural Network optimized with Arithmetic optimization algorithm for lung disease detection using chest X-ray images. Biomed. Signal Process. Control.

[16] Yadav P., Menon N., Ravi V., Vishvanathan S. (2021). Lung-GANs: Unsupervised representation learning for lung disease classification using chest CT and X-ray images. IEEE Trans. Eng. Manag..

[17] Sulaiman A., Anand V., Gupta S., Asiri Y., Elmagzoub M., Reshan M.S.A., Shaikh A. (2023). A Convolutional Neural Network Architecture for Segmentation of Lung Diseases Using Chest X-ray Images. Diagnostics.

[18] Ahmed I., Ahmad M., Jeon G. (2022). Integrating digital twins and deep learning for medical image analysis in the era of COVID-19. Virtual Real. Intell. Hardw..

[19] Tai Y., Zhang L., Li Q., Zhu C., Chang V., Rodrigues J.J., Guizani M. (2022). Digital-Twin-Enabled IoMT System for Surgical Simulation Using rAC-GAN. IEEE Internet Things J..

[20] Zhu L., Lu W., Soleimani M., Li Z., Zhang M. (2023). Electrical Impedance Tomography Guided by Digital Twins and Deep Learning for Lung Monitoring. IEEE Trans. Instrum. Meas..

[21] Xing L., Liu W., Liu X., Li X. (2023). An Enhanced Vision Transformer Model in Digital Twins Powered Internet of Medical Things for Pneumonia Diagnosis. IEEE J. Sel. Areas Commun..

[22] Kaggle, Multiclass Chest X-ray Disease Dataset. https://www.kaggle.com/datasets/saifurrahmanshatil/multiclass-chest-xray-disease-dataset.

[23] Kaggle, Lungs Disease Dataset (4 Types). https://www.kaggle.com/datasets/omkarmanohardalvi/lungs-disease-dataset-4-types.

[24] Kaggle, Multi Classe Chest X-ray DATASET(VERSION 2). https://www.kaggle.com/datasets/sourov509/multi-classe-chest-X-ray-datasetversion-2.

[25] Kaggle, Tuberculosis Chest X-rays (Shenzhen). https://www.kaggle.com/datasets/raddar/tuberculosis-chest-xrays-shenzhen/data.

[26] Kaggle, Chest X-rays Tuberculosis from India. https://www.kaggle.com/datasets/raddar/chest-xrays-tuberculosis-from-india.

[27] Kaggle, Balanced Augmented Covid CXR Dataset. https://www.kaggle.com/datasets/tr1gg3rtrash/balanced-augmented-covid-cxr-dataset.

[28] YOLOv8, Roboflow. https://blog.roboflow.com/whats-new-in-yolov8/.

[29] Sikati J., Nouaze J.C. (2023). YOLO-NPK: A Lightweight Deep Network for Lettuce Nutrient Deficiency Classification Based on Improved YOLOv8 Nano. Eng. Proc..

[30] Inui A., Mifune Y., Nishimoto H., Mukohara S., Fukuda S., Kato T., Furukawa T., Tanaka S., Kusunose M., Takigami S. (2023). Detection of elbow OCD in the ultrasound image by artificial intelligence using YOLOv8. Appl. Sci..

